# Supplementary material for: Inhaled Corticosteroids and the Risk of Nontuberculous Mycobacterial Pulmonary Disease in Chronic Obstructive Pulmonary Disease: Findings from a Nationwide Population-Based Study
Source: J Pers Med. 2023 Jun 30;13(7):1088. doi: 10.3390/jpm13071088 (PMC10382049; doi:10.3390/jpm13071088)
Supplement: Supplementary file 1 [file jpm-13-01088-s001.zip › jpm-2444535-supplementary.pdf]

Table S1. Univariate and multivariate analyses to compare the risk of NTM occurrence

	Univariate analysis		Multivariate analysis	
	Hazard ratio(95%CI)	*P* value	Hazard ratio(95%CI)	*P* value
**Non-ICS users**	1.000		1.000	
**ICS users**	1.060(0.932-1.205)	0.373	1.121(0.950-1.323)	0.176
**ICS cumulative dose** (μg)				
0 μg	1.000		1.000	
0 – 15,000 μg	0.791(0.611-1.023)	0.074	0.761(0.588-0.986)	0.039
15,001 – 60,000 μg	0.943(0.758-1.173)	0.599	0.933(0.750-1.160)	0.532
60,001 – 225,00 μg	0.909(0.732-1.130)	0.391	0.855(0.688-1.063)	0.159
> 225,000 μg	1.459(1.242-1.714)	<.001	1.200(0.950-1.323)	0.050
**ICS daily dose**				
No	1.000		1.000	
Low	0.908(0.773-1.067)	0.242	0.967(0.798-1.171)	0.728
Medium	1.181(1.001-1.394)	0.049	1.229(1.008-1.499)	0.041
High	1.728(1.335-2.239)	<0.001	1.637(1.241-2.160)	<0.001
**Types of ICS**				
No	1.000		1.000	
Fluticasone propionate	1.057(0.908-1.229)	0.475	1.134(0.937-1.372)	0.197
Budesonide	1.066(0.868-1.310)	0.543	1.169(0.929-1.473)	0.183
Beclomethasone	1.055(0.760-1.646)	0.749	1.056(0.746-1.494)	0.758
Ciclesonide	1.351(0.910-2.006)	0.136	1.351(0.906-2.014)	0.140
Fluticasone furoate	0.945(0.685-1.303)	0.729	0.861(0.614-1.208)	0.387
